# Carboxy Terminal Tail of Polycystin-1 Regulates Localization of TSC2 to Repress mTOR

**DOI:** 10.1371/journal.pone.0009239

**Published:** 2010-02-16

**Authors:** Ruhee Dere, Patricia D. Wilson, Richard N. Sandford, Cheryl Lyn Walker

**Affiliations:** 1 Department of Carcinogenesis, University of Texas M.D. Anderson Cancer Center, Smithville, Texas, United States of America; 2 Department of Pediatrics, Children's Research Institute, Medical College of Wisconsin, Milwaukee, Wisconsin, United States of America; 3 Department of Medical Genetics, University of Cambridge, Cambridge, United Kingdom; Roswell Park Cancer Institute, United States of America

## Abstract

Autosomal dominant polycystic kidney disease (ADPKD) is a commonly inherited renal disorder caused by defects in the *PKD1* or *PKD2* genes. ADPKD is associated with significant morbidity, and is a major underlying cause of end-stage renal failure (ESRF). Commonly, treatment options are limited to the management of hypertension, cardiovascular risk factors, dialysis, and transplantation when ESRF develops, although several new pharmacotherapies, including rapamycin, have shown early promise in animal and human studies. Evidence implicates polycystin-1 (PC-1), the gene product of the *PKD1* gene, in regulation of the mTOR pathway. Here we demonstrate a mechanism by which the intracellular, carboxy-terminal tail of polycystin-1 (CP1) regulates mTOR signaling by altering the subcellular localization of the tuberous sclerosis complex 2 (TSC2) tumor suppressor, a gatekeeper for mTOR activity. Phosphorylation of TSC2 at S939 by AKT causes partitioning of TSC2 away from the membrane, its GAP target Rheb, and its activating partner TSC1 to the cytosol via 14-3-3 protein binding. We found that TSC2 and a C-terminal polycystin-1 peptide (CP1) directly interact and that a membrane-tethered CP1 protects TSC2 from AKT phosphorylation at S939, retaining TSC2 at the membrane to inhibit the mTOR pathway. CP1 decreased binding of 14-3-3 proteins to TSC2 and increased the interaction between TSC2 and its activating partner TSC1. Interestingly, while membrane tethering of CP1 was required to activate TSC2 and repress mTOR, the ability of CP1 to inhibit mTOR signaling did not require primary cilia and was independent of AMPK activation. These data identify a unique mechanism for modulation of TSC2 repression of mTOR signaling via membrane retention of this tumor suppressor, and identify PC-1 as a regulator of this downstream component of the PI3K signaling cascade.

## Introduction

Autosomal dominant polycystic kidney disease (ADPKD), is characterized by the progressive, bilateral enlargement of the kidneys due to multiple cysts that arise from the tubular epithelial cells of the nephron [1], [2]. ADPKD has an incidence of 1 in 500 to 1 in 1000 live births and is the leading cause of end-stage renal disease (ESRD) in the US. Although ADPKD is primarily characterized by renal cysts, it is a systemic disorder, resulting in epithelial cysts in multiple organs including the liver and pancreas [3], [4]. Non-cystic manifestations include hypertension, cardiac valve abnormalities, and intracranial aneurysms [5]. Currently, treatment for advanced ADPKD is limited to renal transplantation or life-long hemodialysis [4]. Almost 85% of the ADPKD cases result from mutations in the *PKD1* gene on chromosome 16 that encodes polycystin-1 [6], whereas mutations in the *PKD2* gene on chromosome 4 encoding polycystin-2, are responsible for the remaining 15% of the cases [7], [8].

Polycystin-1 (PC-1) is a large (4303 aa) integral membrane glycoprotein (molecular mass ∼460 kDa), which includes a long (∼3000 aa) N-terminal extracellular domain, 11 trans-membrane domains and a short (∼200 aa) intracellular C-terminal tail [9], [10], [11], [12]. PC-1 interacts via its coiled-coil domain with polycystin-2 (PC-2), also an integral membrane protein, to act as a calcium permeable cation channel [13]. Additionally, PC-1 has been localized to cell-cell junctions where it modulates cell adhesion [14], [15], and at sites of cell-matrix interactions [16]. PC-1 has also been localized to the primary cilium of renal epithelial cells, where it is thought to be involved in ciliary mechanotransduction [17]. The C-terminal tail of PC-1 has been reported to regulate various signaling pathways [4] including Wnt signaling pathway [18], AP-1 transcription factor complex signaling [19], [20] and more recently, STAT6 signaling to stimulate STAT6-dependent gene expression [21].

Accumulating evidence suggests that PC-1 might have a functional link to the tuberous sclerosis complex 2 (TSC2) tumor suppressor [22], [23], [24]. TSC2 lies at the epicenter of signal integration in the conserved mTOR signaling cascade, which regulates protein synthesis and cell growth [25], [26]. The *TSC2* gene is mutated in tuberous sclerosis complex (TSC), a systemic disorder characterized by benign hamartomas especially of the kidney [27]. The heterodimeric TSC2/TSC1 complex has a highly specific GAP (GTPase activating protein) activity towards Rheb (Ras homolog enriched in brain), a major regulator of mTORC1 (mammalian target of rapamycin complex 1) [28]. Activated mTORC1 phosphorylates and activates its down-stream effectors ribosomal S6 kinases - S6K1 and S6K2 and eIF4E (eukaryotic initiation factor 4E)-binding proteins, 4E-BP1 and 4E-BP2 to stimulate protein synthesis and proliferation [29], [30], [31].

Studies have shown aberrant activation of mTOR in several rodent models of polycystic kidney disease [22], [32], [33] and treatment with rapamycin has been shown to alleviate cyst enlargement in murine models [34], [35], [36]. Furthermore, deletion of PKD1 and TSC2 in a contiguous gene deletion syndrome, exhibits a severe PKD phenotype [37] further suggesting that these two proteins may be involved in a common pathway. TSC2 is required for the normal trafficking of PC-1 from the Golgi to the membrane [23] and more recently, PC-1 was also shown to interact with TSC2 [22], establishing a functional link between these two proteins. In a recent study, PC-1 was found to inhibit ERK-mediated phosphorylation of TSC2 at S664 [24] in both MDCK (Madin-Darby canine kidney) and MEF (mouse embryonic fibroblast) cells.

We have used a human model system to elucidate the mechanism by which PC-1 regulates the mTOR pathway and found that PC-1 modulates phoshatidylinositol 3-kinase (PI3K)/AKT signaling to TSC2 to repress mTOR. AKT phosphorylates TSC2 at several sites including S939, which results in the membrane partitioning of TSC2 to the cytosol, away from its GAP target Rheb and its activating partner TSC1 via binding of 14-3-3 proteins [38]. We found that the C-terminal tail of human polycystin-1 (CP1) directly interacts with TSC2 and when membrane-tethered, prevents the inactivating phoshorylation of TSC2 at S939, thus retaining TSC2 at the membrane. This membrane retention of TSC2 enhances TSC2 repression of mTOR signaling in human cells. Importantly, the ability of CP1 to protect TSC2 from AKT phosphorylation and enhance mTOR repression occurred in the absence of primary cilia, indicating that repression of mTOR occurs independently of CP1 localization to this organelle.

## Results

### Activation of mTOR Signaling in Human ADPKD

**Figure 1:**
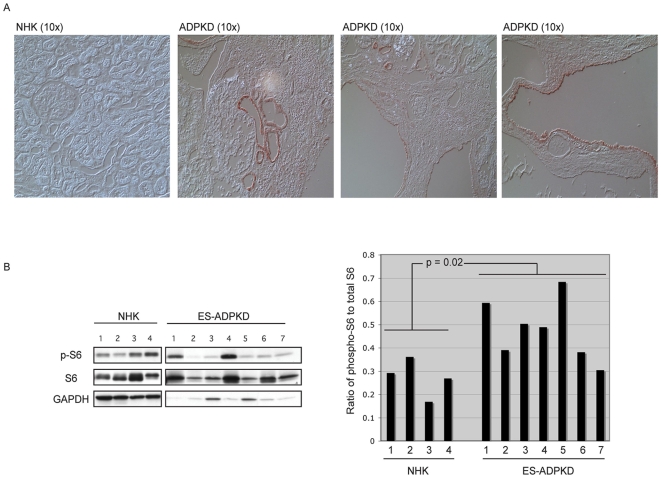
Dysregulation of mTOR in human ADPKD patients. **A.** Normal human kidney (NHK) and ES-ADPKD kidney tissues were analyzed by immunohistochemistry using anti-phospho-S6 antibody (stained red). The sections were visualized using Differential Interference Contrast (DIC) microscopy. Representative images at 10x magnification are shown. **B.** Western blot analysis of tissue lysates from 4 NHK and 8 ES-ADPKD kidneys, immunoblotted with anti-phospho-S6 antibody (left). The autoradiographs were quantified and plotted as a ratio of phosphorylated S6 to total S6 (right). A statistically significant (p≤0.05) increase in phosphorylation of S6 was observed in ES-ADPKD kidneys compared to the NHKs.

Limited evidence from recent studies has suggested a role for loss of polycystin-1 (PC-1) in dysregulation of mTOR signaling in human ADPKD [22], [33]. We confirmed elevated mTOR signaling in cysts of kidneys from patients with end-stage autosomal dominant polycystic kidney disease (ES-ADPKD) relative to normal kidneys (NHK) from unaffected individuals. Immunohistochemistry from 20 ES-ADPKD kidneys and 8 NHK (in the age range of 42–59 years) demonstrated that in many cysts, epithelial cells lining the cysts exhibited elevated phospho-S6 staining compared to the normal epithelial cells in unaffected NHKs (Figure 1A). Consistent with previous reports [33], however, immunoreactivity for phospho-S6 was heterogeneous, with both phospho-S6 positive and phospho-S6 negative cysts observed in affected kidneys. Immunohistochemistry data were confirmed by western analysis of tissue from 8 ES-ADPKD kidneys and 4 NHK's. Even though cystic kidneys from ES-ADPKD patients contain a mixture of phospho-S6 positive and negative cysts as well as apparently unaffected epithelial cells, and stromal cells, western analysis was able to detect elevated levels of phospho-S6 indicative of mTOR activation in ES-ADPKD kidneys relative to NHK (Figure 1B) (two-tailed unpaired Student's t-test) (p≤0.02). Thus, for at least a subpopulation of cysts, loss of PC-1 and development of cysts correlated with activation of mTOR signaling.

### The C-Terminal Tail of Polycystin-1 (CP1) Represses mTOR Signaling

**Figure 2:**
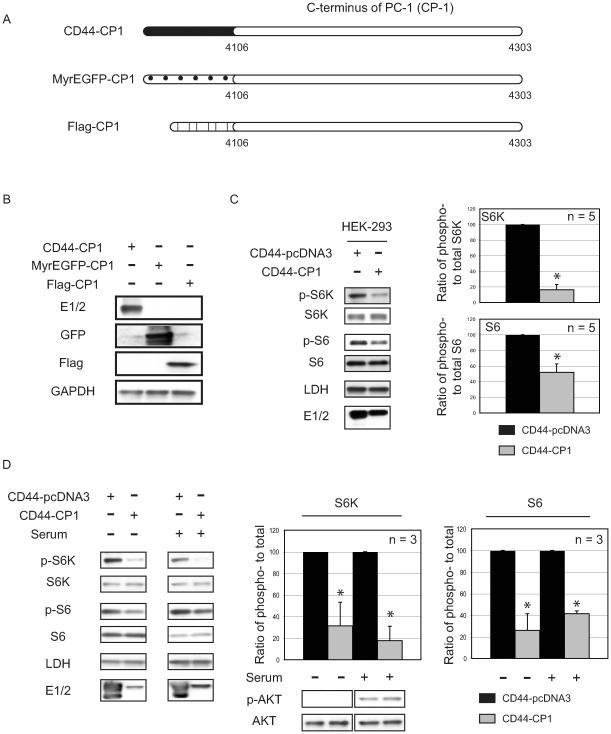
The C-terminal tail of human polycystin-1 (CP1) represses mTOR signaling. **A.** Schematic of the constructs used in the study. CP1 (aa 4106 – aa 4303) was tagged with either membrane-bound CD44, myristolylated EGFP or soluble Flag tag. **B.** Expression of the tagged-CP1 constructs transfected into HEK-293 cells was determined by immunoblotting using E1/2, anti-GFP and anti-Flag antibodies. **C.** Protein lysates generated from HEK-293 cells (n = 5) expressing CD44-pcDNA3 (vector control) or CD44-CP1 were analyzed using the indicated antibodies (left). The autoradiographs were quantified and plotted as a ratio of phospho- to total protein (S6K and S6), where the vector control (CD44-pcDNA3, black bars) and CD44-CP1 (gray bars) were normalized to 100. **D.** HEK-293 cells were transfected with the vector control (CD44-pcDNA3) or CD44-CP1 and treated as indicated (n = 3). Protein lysates generated from these cells were analyzed by immunoblotting using the indicated antibodies (left). The graphs denote the ratio of phospho- to total protein (S6K and S6) (right) where the vector control (CD44-pcDNA3, black bars) and CD44-CP1 (gray bars) ratios were normalized to 100. All blots shown are indicative of a single representative experiment and an * denotes a statistically significant difference (p≤0.05) between vector control (CD44-pcDNA3) and CD44-CP1.

To explore a potential mechanism by which loss of PC-1 might disrupt mTOR signaling in cystic epithelial cells, we focused on the C-terminal, cytoplasmic tail of PC-1. The large size of PC-1 (4303 aa) limits its transfectability in transient transfection assays, therefore the cytosolic tail of PC-1 has been used to investigate its function [19], [39], [40], [41], [42]. As shown in Figure 2A, we utilized the C-terminal tail of human polycystin-1 (CP1) (aa 4106 – aa 4303) tagged with either the extracellular and transmembrane region of CD44-, an integral membrane peptide (CD44-CP1), or a myristoylation signal (MyrEGFP-CP1) to express membrane-tethered CP1, or Flag (Flag-CP1) to express soluble CP1. Expression of CD44-, MyrEGFP- and Flag-CP1 following transient transfection into HEK-293 (human embryonic kidney) epithelial cells or hTERT RPE-1 (human retinal pigment epithelial) cells confirmed that high levels of both membrane tethered and soluble CP1 were expressed from these constructs (Figure 2B and data not shown).

The ability of CP1 to regulate mTOR signaling in human cells was demonstrated by decreased phosphorylation of the mTOR target S6K and its downstream effector S6 in CD44-CP1 expressing HEK-293 cells compared to vector (CD44-pcDNA3) control cells expressing CD44 alone (Figure 2C, left). Phosphorylation of S6K and S6 decreased significantly, by 90% (p≤0.001, n = 5) and 49% (p≤0.001, n = 5), respectively as shown in Figure 2C (right). Similar data were obtained in retinal epithelial hTERT RPE-1 cells (data not shown), confirming the ability of CP-1 to regulate mTOR signaling in human cells.

To investigate if CP1 was able to repress mTOR signaling under conditions of mitogen stimulation that acutely activates PI3K/AKT and mTOR, HEK-293 cells were starved, and then stimulated for 1 hour with serum. As shown in Figure 2D, CD44-CP1 significantly repressed mTOR signaling even under conditions of mitogen stimulation when AKT was activated as assessed by phospho-AKT (S473) levels. In serum-starved cells expressing CD44-CP1, phosphorylation of S6K and S6 was significantly lower, decreased by 69% (p≤0.01, n = 3) and 75% (p≤0.01, n = 3), respectively over vector control (CD44-pcDNA3) (Figure 2D, right). Upon stimulation of PI3K/AKT, CD44-CP1 repression of mTOR was even more pronounced, with 83% (S6K, p≤0.01, n = 3) and 60% (S6, p≤0.001, n = 3) inhibition observed (Figure 2D, right). These data indicate that CP1 is able to regulate mTOR signaling in human epithelial cells in both the absence and presence of a strong mitogenic signal.

**Figure 3:**
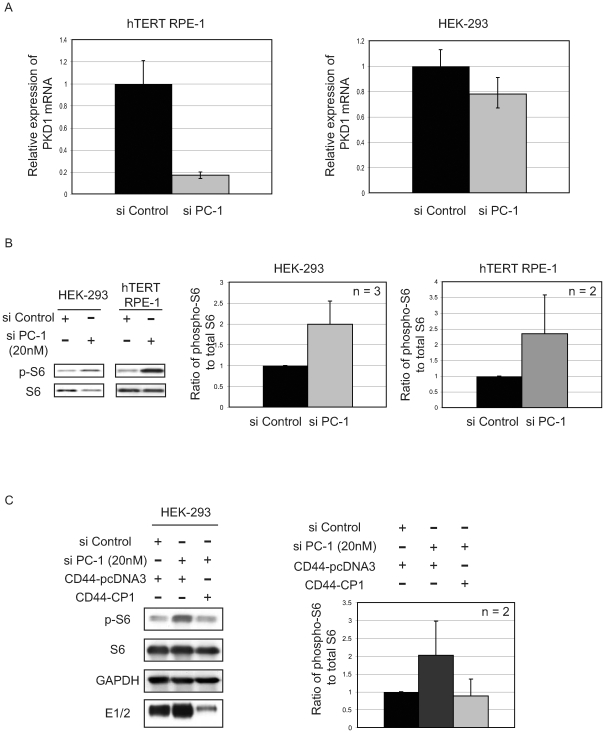
Polycystin-1 knockdown results in the activation of mTOR signaling *in vitro*. **A.** RT-PCR analysis of mRNA collected from hTERT RPE-1 and HEK-293 cells expressing human PC-1 siRNA (20 nM). The relative expression of PC-1 is shown in the presence of scrambled (control, black bars) and human PC-1 (gray bars) siRNA, normalized to 1. All RT-PCR reactions were performed in triplicate. **B.** Cell lysates collected from HEK-293 and hTERT RPE-1 cells 48 hours following transfection of scrambled and PC-1 siRNA were examined by immunoblotting using the specified antibodies (left). The blots shown indicate a single representative experiment. The graphs (right) indicate the ratio of phospho- to total S6 where the scrambled (black bars) and PC-1 siRNA (gray bars) ratios were normalized to 1. **C.** Protein lysates from HEK-293 and hTERT RPE-1 cells, transfected with control or human PC-1 siRNA and co-expressing CD44-pcDNA3 (vector control) or CD44-CP1, were analyzed by immunoblotting using the indicated antibodies (left). The western blots are representative of two independent experiments. The autoradiographs were quantified and plotted (right) as a ratio of phospho- to total S6. The control siRNA (with CD44-pcDNA3) is depicted as a black bar and PC-1 siRNA with either CD44-pcDNA3 or CD44-CP1 is shown as gray bars.

Following our observation that expression of exogenous CP1 was sufficient to repress mTOR signaling, we next determined whether endogenous PC-1 regulated mTOR signaling in human epithelial cells, using small interfering RNA's (siRNA) to knockdown endogenous PC-1 in HEK-293 and hTERT RPE-1 cells. Using RT-PCR analysis to determine efficiency of PC-1 knockdown, we were able to achieve an 80% and 20% knockdown of PC-1 mRNA in hTERT RPE-1 and HEK-293 cells, respectively (Figure 3A). Although higher concentrations of siRNA (>20nM) resulted in more efficient knockdowns, these were cytotoxic, and therefore for our studies we chose the least toxic concentration of PC-1 siRNA, which was 20 nM. Under these conditions we found that mTOR signaling increased at least 2-fold as assessed by phosphorylation of S6, as shown in Figure 3B, in both HEK-293 (n = 3) and hTERT RPE-1 (n = 2) cells. Interestingly, although PC-1 expression was reduced by only 20% (Figure 3A), likely due to effective knockdown of PC-1 in only a portion of HEK-293 cells, increased activation of the mTOR pathway was readily detectable (Figure 3B) suggesting that a large increase in , mTOR signaling occurred in HEK-293 cells depleted of PC-1.

To confirm that elevated mTOR signaling was due to loss of PC-1 expression and not an off-target effect, CD44-CP1 was re-expressed following endogenous PC-1 knockdown to rescue mTOR repression. The smart-pool human PC-1 siRNA sequence was compared to CD44-CP1 to verify lack of sequence homology and ensure that CD44-CP1 would be expressed in conjunction with the PC-1 knockdown (data not shown). HEK-293 cells were transfected with PC-1 siRNA and subsequently transfected with CD44-pcDNA3 (control) or CD44-CP1 and mTOR signaling was assessed by western analysis for phosphorylation of S6. HEK-293 PC-1 siRNA transfected cells, co-transfected with control vector (CD44-pcDNA3) exhibited a 2-fold elevation in mTOR signaling relative to si-control (scrambled sequence) co-transfected with CD44-pcDNA3 (Figure 3C, right). However, knocking down endogenous PC-1 and expressing CD44-CP1 abrogated the effect of PC-1 siRNA on mTOR signaling, decreasing dramatically phosphorylation of S6 to basal levels (Figure 3C). Thus, PC-1, and likely its cytoplasmic tail, modulates mTOR signaling *in vitro*, consistent with *in vivo* data demonstrating mTOR activation in cysts of ADPKD patients.

### CP1 Repression of mTOR Requires TSC2, but Not Primary Cilia or AMPK

PC-1 is localized to the primary cilia and is implicated in flow-induced mechanotransduction, and mutations in PC-1 are linked to the ciliary defects associated with ADPKD [17]. However, HEK-293 cells, which showed inhibition of mTOR signaling in response to expression of CD44-CP1 (Figure 2C and 2D), were non-ciliated (≤2%), suggesting that repression of mTOR signaling was a cilia-independent function of CP1. Similarly, inhibition of mTOR signaling observed in hTERT RPE-1 cells, occurred under non-ciliated conditions when≤5% of cells were ciliated, as confirmed by staining with cilia-specific anti-acetylated alpha-tubulin (Figure S1). Thus, the inhibition of the mTOR signaling cascade by CP1 did not require primary cilia, and occurs via cilia-independent pathway(s).

**Figure 4:**
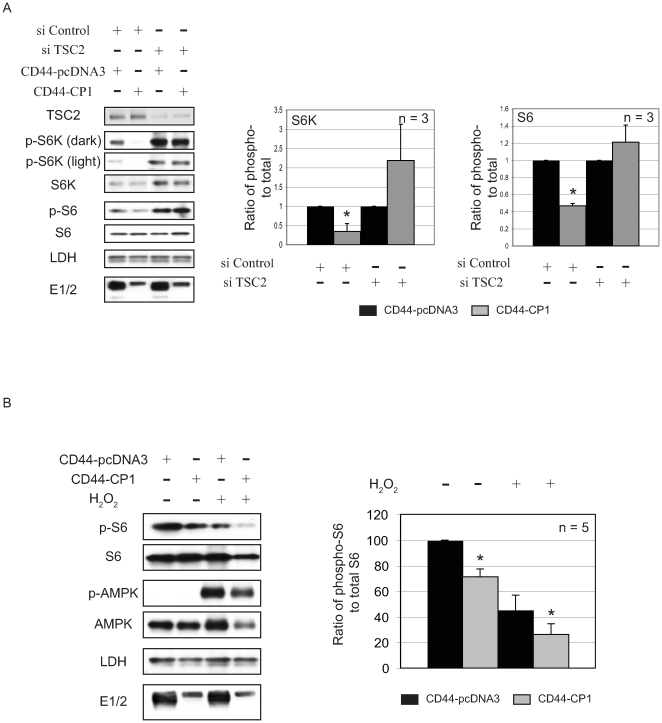
CP1 inhibits mTOR via a TSC2-dependent mechanism, distinct from AMPK activation of TSC2. **A.** HEK-293 cells transfected with scrambled or TSC2 siRNA, co-expressing CD44-pcDNA3 or CD44-CP1 were used to generate protein lysates that were analyzed by immunoblotting using the indicated antibodies. Blots represent a single experiment (n = 3) and the graph is a ratio of phospho- to total protein. The ratios for CD44-pcDNA3 (vector control, black bars) and CD44-CP1 (gray bars) were normalized to 1 as shown. **B.** Protein lysates generated from HEK-293 cells expressing CD44-pcDNA3 (control) or CD44-CP1, treated with 0.4 mM H_2_O_2_ were analyzed by immunoblotting with the indicated antibodies. The autoradiographs were quantified and plotted as a ratio of phospho-S6 to total S6 for the conditions indicated. Representative blots for a single experiment are shown (n = 5). The * denotes a statistically significant difference between the vector control (CD44-pcDNA3, black bars) and CD44-CP1 (gray bars) (p≤0.05).

TSC2 is an important gatekeeper of mTOR activity, negatively regulating mTOR signaling [28], prompting us to examine TSC2 as a possible mediator of PC-1 regulation of mTOR. As shown in Figure 4A, in HEK-293 cells transfected with TSC2 siRNA, endogenous TSC2 levels were decreased by more than 80% compared to cells transfected with scrambled siRNA, and was associated with increased mTOR signaling (Figure 4A, compare lanes 1 and 3). In cells expressing control (scrambled) siRNA and CD44-CP1, mTOR was repressed (Figure 4A, lane 2). However, in cells depleted of TSC2 (TSC2 knockdown cells), CD44-CP1 was unable to repress mTOR signaling (Figure 4A, lane 4), indicating that CP1 regulation of mTOR was TSC2-dependent.

TSC2 is itself regulated by several signaling pathways that regulate mTOR, including the energy sensing AMP-activated protein kinase (AMPK) pathway. AMPK activates the tumor suppressor function of TSC2, leading us to investigate whether AMPK was potentially mediating PC-1 activation of TSC2. However, AMPK was not activated in cells expressing CD44-CP1 under conditions where CP1 repressed mTOR signaling (Figure 4B). Furthermore, mTOR signaling could be further repressed by CP1 under conditions in which AMPK was activated and mTOR was repressed by TSC2. HEK-293 cells expressing either control (CD44-pcDNA3) or CD44-CP1, were treated with 0.4 mM H_2_O_2_ to activate AMPK [43], confirmed by immunoblotting using a phospho-specific AMPK (T172) antibody as shown in Figure 4B (left). In cells with activated AMPK, CD44-CP1 further augmented the repression of mTOR when compared to vector control (CD44-pcDNA3). On average a significant decrease was observed in the ratio of phospho-S6 to total S6 in non-treated cells (cells grown in normal growth media) expressing CD44-CP1 compared to the control (p≤0.01, n = 5) (Figure 4B, right) as well as cells treated with H_2_O_2_ (p≤0.01, n = 5). These data indicate that CD44-CP1 repression of mTOR does not require AMPK activation of the tumor suppressor function of TSC2, and that CP1 can augment activation of TSC2 beyond that achieved by AMPK activation.

### CP1 Protects TSC2 from Inactivation by AKT

**Figure 5:**
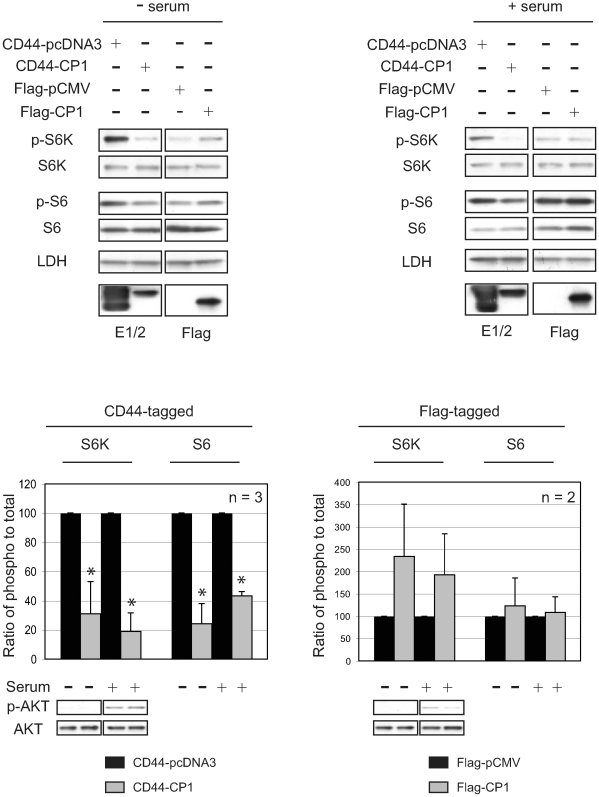
Membrane tethering of CP1 is required for its regulation of mTOR. HEK-293 cells were transfected with CD44-pcDNA3 (vector control), CD44-CP1, Flag-pCMV (vector control) or Flag-CP1 and treated as indicated. Protein lysates were analyzed by immunoblotting with the indicated antibodies (left). The graphs indicate the ratios of phospho- to total protein for the vector controls (CD44-pcDNA3 or Flag-pCMV, black bars) and CD44-CP1 or Flag-CP1 (gray bars). Blots represent a single experiment (n = 3 with CD44-tagged constructs, and n = 2 with Flag-tagged constructs) under the indicated conditions and the ratios were normalized to 100. An * denotes a statistically significant (p≤0.05) difference between CD44-CP1 compared to its vector control (CD44-pcDNA3).

TSC2 regulation of mTOR requires membrane localization of this tumor suppressor [38], where it co-localizes with its GAP target Rheb and its activation partner TSC1. To determine if membrane localization of CP1 impacted its ability to activate TSC2, we compared the ability of membrane-tethered (CD44-CP1) and soluble (Flag-CP1) CP1 to repress mTOR signaling. In the absence of serum, mTOR was repressed by membrane-tethered CD44-CP1, as indicated by the significantly decreased phosphorylation of S6K and S6 (Figure 5) by 70% (p≤0.05, n = 3) and 76% (p≤0.05, n = 3) respectively, when compared to the control (CD44-pcDNA3). The ability of membrane-localized CP1 to repress mTOR was confirmed using myristoylated-EGFP-CP1 (myrEGFP-CP1), which reduced phosphorylation of S6K by more than 50% (data not shown). In contrast, soluble CP1 exhibited no repression of the mTOR pathway in quiescent or serum-stimulated cells. As shown in Figure 5, rather than a decrease as observed for membrane-tethered CP1, the ratio of phospho- to total S6K increased almost 2-fold, in quiescent and serum-stimulated cells expressing Flag-CP1 compared to the control (Flag-pCMV) (Figure 5, right). Interestingly, in serum-starved cells, expression of soluble CP1 actually increased mTOR signaling, suggesting that CP1 that could not localize to the membrane inhibited the ability of TSC2 to repress mTOR. Thus, CP1 functionality (i.e. repression of mTOR) required its membrane localization.

**Figure 6:**
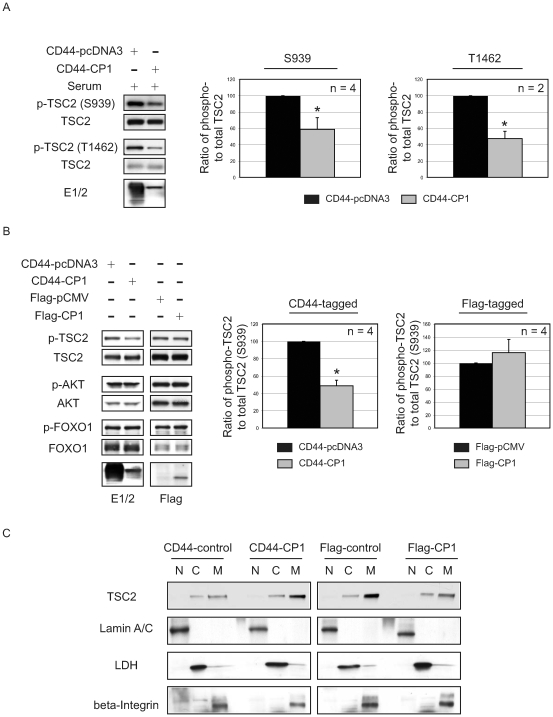
CP1 represses mTOR, by protecting TSC2 from its inactivating phosphorylation by AKT. **A.** Cell lysates obtained from HEK-293 cells expressing CD44-pcDNA3 (vector control) and CD44-CP1, treated with serum for 1h following serum starvation, were immunoblotted using the indicated antibodies. Blots from a single representative experiment are shown for phosphorylation at S939 (n = 4) and T1462 (n = 2). The graph on the right indicates the ratio of phospho- to total TSC2 at residues S939 and T1462. An * denotes a statistically significant difference between vector control (CD44-pcDNA3, black bars) and CD44-CP1 (gray bars) (p≤0.05). **B.** Cell lysates obtained from HEK-293 cells grown in normal growth media containing 10% FBS, and expressing CD44-pcDNA3 (vector control), CD44-CP1, Flag-pCMV (vector control) or Flag-CP1 were analyzed using the indicated antibodies. Blots from a single representative experiment are shown (n = 4). The graph on the right denotes the ratio of phospho- to total TSC2 (S939) for both CD44-tagged and Flag-tagged constructs. Black bars indicate the controls whereas the gray bars indicate either CD44-CP1 or Flag-CP1. The * denotes a statistically significant difference between CD44-pcDNA3 (vector control) and CD44-CP1 (p≤0.001). **C.** Subcellular fractionation of HEK-293 cells expressing CD44-pcDNA3 (vector control), CD44-CP1, Flag-pCMV (vector control), or Flag-CP1. A representative blot (n = 3) showing the separated fractions analyzed using anti-TSC2, anti-lamin A/C (nuclear maker), anti-LDH (cytosolic marker), and anti-beta-integrin (membrane marker). N – nuclear, C – cytosolic, M – membrane fraction.

As shown in Figure 2C, CP-1 was able to repress mTOR even under conditions of mitogen stimulation when AKT is activated. In response to mitogenic stimulation, AKT phosphorylates TSC2 on multiple residues, including S939, which causes TSC2 to be partitioned away from the membrane by 14-3-3 proteins, thereby relieving the inhibitory effect of TSC2 on Rheb and mTOR [38]. Concordant with mTOR repression, phosphorylation of TSC2 at S939 was significantly reduced in cells expressing CD44-CP1 compared to vector control (CD44-pcDNA3) when cells were stimulated with serum for 1 h (Figure 6A). Similarly, phosphorylation of TSC2 at T1462, an AKT phosphorylation site not involved in membrane localization of TSC2, was also decreased (Figure 6A). Quantitation of the ratio of phospho- to total TSC2 (Figure 6A, right), indicated a similar reduction of 40% and 60% (p≤0.01) in AKT phosphorylation at S939 and T1462, respectively. In contrast to the cells transfected with functional CD44-CP1 (Figure 6B), cells expressing inactive Flag-CP1 showed no difference in the phosphorylation of TSC2 at S939 (Figure 6B), indicating that active, membrane-bound CP1 could protect TSC2 from its inactivating phosphorylation at this site. Decreased TSC2 phosphorylation was not due to decreased AKT activity in response to expression of CP1, as AKT remained active, as evidenced by continued AKT phosphorylation at S473, and verified by sustained phosphorylation of FoxO1, a downstream target of AKT (Figure 6B) in CD44-CP1 expressing cells.

AKT phosphorylation of TSC2 at S939 and S981 results in the binding of 14-3-3 proteins to TSC2, leading to the partitioning of TSC2 away from the membrane into the cytosol and inactivating this tumor suppressor [38]. To determine if CD44-CP1 protection of TSC2 from phosphorylation by AKT resulted in enhanced membrane retention of TSC2, subcellular fractionation of HEK-293 cells expressing CD44-CP1 was performed. In the presence of CD44-CP1, enhanced membrane localization of endogenous TSC2 was observed compared to cells expressing control CD44-pcDNA3 (Figure 6C). In contrast, in cells expressing non-functional Flag-CP1, less TSC2 is localized to the membrane and the ratio of TSC2 in the membrane *vs.* cytosol was greatly reduced (note overall levels of TSC2 were also reduced due to the instability of cytosolic TSC2 [44]. Thus, CP-1 protection of TSC2 from inactivating phosphorylation by AKT correlated with retention of TSC2 at the membrane (Figure 6C).

**Figure 7:**
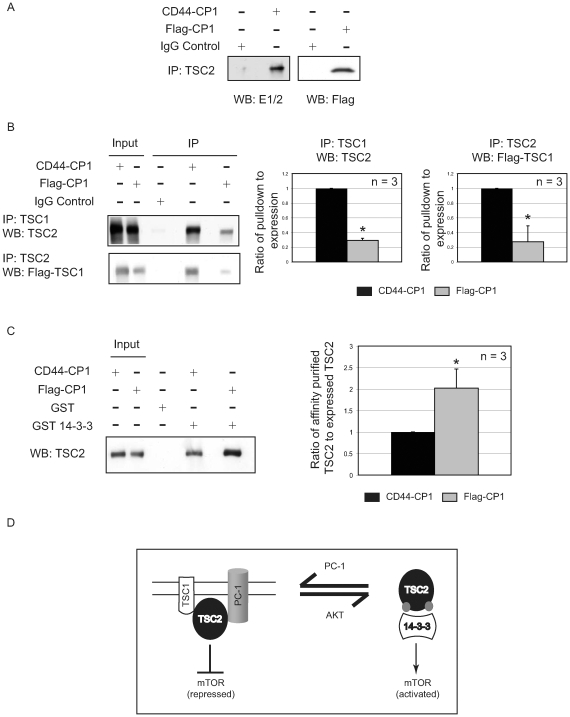
Interaction of tuberin with hamartin and 14-3-3 in cells expressing functional and non-functional CP1. **A.** Lysates generated from HEK-293 cells, expressing functional, membrane-bound CD44-CP1 and non-functional, soluble Flag-CP1, were used for immunoprecipitation with anti-TSC2 and the immunoprecipitates further analyzed by immunoblotting with E1/2 and anti-Flag antibodies. **B.** Whole cell lysates from HEK-293 cells expressing either CD44-CP1 or Flag-CP1 were used for immunoprecipitation with anti-TSC2 or anti-TSC1 antibodies, and the immunoprecipitates analyzed by immunoblotting with anti-Flag (to detect Flag-TSC1), or anti-TSC2, respectively. Blots from a single representative experiment are shown (n = 3). The graph on the right shows the ratio of pull down to the expression of Flag-TSC1 and TSC2 as indicated. Quantification of blots from both immunoprecipitation assays showed a statistically significant difference (*) in the interaction between TSC2 and TSC1, in cells expressing CD44-CP1 (black bars) and Flag-CP1 (gray bars). **C**. Protein lysates from HEK-293 cells expressing CD44-CP1 or Flag-CP1 were affinity purified using GST-14-3-3 or GST alone and immunoblotted with an anti-TSC2 antibody. The immunoblot for a single representative experiment is shown (n = 3). The graph on the right depicts the ratio of affinity-purified TSC2 to total endogenous TSC2 in cells expressing CD44-CP1 (black bar) or Flag-CP1 (gray bar) and the * denotes a statistically significant difference between the two. **D**. Model for polycystin-1 inhibition of mTOR signaling via TSC2.

Interestingly, although only membrane-tethered CP1 could repress mTOR, this was not due to an inability of Flag-CP1 to directly interact with TSC2. As shown in Figure 7A, immunoprecipitation assays revealed that both membrane-bound CD44-CP1 and soluble Flag-CP1 were proficient in binding TSC2. However, only CD44-CP1, in addition to enhancing membrane retention of TSC2, significantly enhanced the interaction between TSC2 and TSC1, as determined by immunoprecipitation assays where TSC1 was co-immunoprecipitated with TSC2 (Figure 7B, lanes 4 and 5) (n = 3). Similar results were obtained with the reverse immunoprecipitation, where TSC2 was immunoprecipitated with TSC1 (Figure 7B, lanes 4 and 5). After normalization, we observed between a 2 - 3-fold increase in interaction between TSC1 and TSC2 in cells expressing CD44-CP1 *vs*. Flag-CP1 (p≤0.05, n = 3) (Figure 7B, right).

Retention of TSC2 at the membrane in cells expressing functional CD44-CP1, suggested that binding of TSC2 to 14-3-3 proteins was reduced, commensurable with decreased phosphorylation at S939. This was confirmed in HEK-293 cells expressing endogenous TSC2 and, either functional CD44-CP1 or non-functional Flag-CP1. GST-14-3-3 affinity purification, revealed enhanced binding of TSC2 to 14-3-3 proteins in the presence of non-functional Flag-CP1 as compared to functional CD44-CP1 as shown in Figure 7C. When the amount of affinity purified TSC2 was normalized to expression of endogenous TSC2, an almost 2-fold enhanced binding of TSC2 to 14-3-3 was observed in Flag-CP1 expressing cells relative to cells expressing CD44-CP1 (Figure 7C, right) (p≤0.05, n = 3). Thus, as shown in Figure 7D, membrane-tethered CP1 enhanced TSC2 retention at the membrane and protected this tumor suppressor from AKT phosphorylation to enhance TSC2 repression of mTOR.

## Discussion

Accumulating evidence implicates PC-1 and TSC2 in the mTOR pathway [22], [24], [33] suggesting that both PKD and TSC may share a common pathway of renal cystogenesis. We have demonstrated that the short intracellular C-terminal tail of polycystin-1 (CP1) when membrane-bound, enhances TSC2-mediated repression of mTOR signaling in human cells. CP1's ability to interact with and protect TSC2 from inactivating phosphorylation by AKT retained TSC2 at the membrane, where it was active and able to exert its tumor suppressor function to repress mTOR. Importantly, regulation of TSC2 by CP1 appears to be distinct from AMPK activation of TSC2, and does not require localization of CP1 to primary cilia. Thus, we have elucidated a mechanism by which PC-1 regulates mTOR, a critical kinase that controls cell growth and proliferation.

Previous reports showed activation of the mTOR pathway in human ADPKD patients by immunohistological staining using tissue from ADPKD patients [22], [33]. We have confirmed this observation and demonstrated that knocking down endogenous PC-1 in human epithelial cells *in vitro* also elevated mTOR signaling. We observed activation of the mTOR pathway even under conditions of a 20% knockdown (HEK-293 cells). Importantly, a dramatic increase in the phosphorylation of S6K and S6 detectable by western analysis under conditions where 20% of the cells have lost PC-1 is sufficiently large so as to be detected even in this heterogeneous population. This is consistent with clinical data, where even though cyst positivity is heterogeneous, positive cysts show dramatic differences in immunoreactivity relative to unaffected tubules. Importantly, what remains to be established is how heterogeneity for loss of PC-1 correlates with the observed heterogeneity for mTOR activation, especially given the likelihood that additional events are required for both cystogenesis and mTOR activation observed in cystic epithelial cells.

AKT phosphorylates TSC2 to inhibit its tumor suppressor function and activate mTOR signaling. We propose a mechanism by which membrane-tethered CP1 suppresses mTOR signaling by altering the subcellular localization of TSC2 (Figure 7D). In the presence of functional, membrane-bound PC-1/CP1 AKT phosphorylation of TSC2 is inhibited, retention of TSC2 at the membrane is increased, as is the interaction between TSC2 and its activating partner TSC1 and concomitantly, binding of 14-3-3 proteins to TSC2 is decreased. The exact mechanism by which the carboxy-terminus of PC-1 protects TSC2 from inactivating phosphorylation by AKT will require further investigation. However, while recent studies have emphasized the importance of localization of PC-1 to the primary cilia [45], [46], our data using non-ciliated cells would suggest that the regulation of mTOR by CP1 is a cilia-independent function of this cystoprotein.

These data may also go a long way toward explaining recent observations by Distefano *et al*. [24], where in spite of observing the anticipated activation of AKT on expressing full-length PC-1, the authors failed to observe an increase in phosphorylation of TSC2 at S939. In fact, over expressing full-length PC-1 may have protected TSC2 from AKT phosphorylation (our data), suggesting that as per our C-peptide, full-length PC-1 also protects TSC2, further validating this model (Figure 7D). In addition to demonstrating that membrane-tethered CP1 altered the subcellular localization of TSC2 to regulate mTOR signaling, our data also revealed that the localization of CP1 was critical for its function. Importantly, soluble CP1 (Flag-CP1) that was unable to localize to the membrane failed to repress the mTOR pathway and in fact activated the signaling cascade. Although functionally deficient, this soluble CP1 was fully capable of interacting with TSC2, suggesting that soluble CP1 could be interacting with TSC2 in the cytosol, thereby interfering with TSC2's ability to localize to the membrane.

Dysregulation of the mTOR pathway has not only been implicated in ADPKD, but in a number of other renal cystic disorders such as ARPKD [47] and several rodent models with mutations in other cystoprotein genes [17], [48]. Although, studies have demonstrated dysregulation of mTOR signaling associated with pathogenesis, the exact mechanisms by which these various cystoprotein gene products modulate this pathway are not understood. Importantly, the PC1-TSC2 model proposed above identifies a mechanism by which PC-1 regulates the mTOR pathway, and provides new data for future studies aimed at investigating the role of other cystoproteins in regulation of mTOR signaling. Given that rapamycin – an mTOR inhibitor, shows efficacy in several rodent models of polycystic kidney disease [22], [34], [49] it would be interesting to determine if these renal cystoproteins act at or upstream of the PC1-TSC2 node of the pathway, implicating PC1-TSC2 as a pathogenic nexus in these disorders. Future experiments elucidating the functionality of TSC2 in other renal cystic disorders will be critical in answering this question. Although promising, rapamycin has been shown to have a variety of adverse side effects [50], [51] and has yet to exhibit dramatic efficacy as a single-agent therapeutic in cancer settings. Additionally, relief of negative feedback to AKT induced by mTOR inhibitors is a potential adverse consequence of treatment with these drugs, especially in the setting of TSC2 deficiency [52], [53], [54], [55]. If TSC2 is at a pathogenic nexus for cystogenesis in the setting of PKD, the potential to upregulate AKT in response to treatment with rapalogs becomes a significant concern. Recently a dual pan class I PI3K/mTOR catalytic small molecule inhibitor NVP-BEZ-235 used to treat TSC2-deficient kidney tumors did not induce the increase in phospho-AKT (S473) levels observed with RAD001 [53], although both drugs were equally effective in suppressing tumor growth. The identification of the role and function of PC-1 in the mTOR pathway provides additional targets, including AKT, whose modulation either singly, or in combination with rapamycin, may contribute to alternative therapeutic strategies in the treatment of ADPKD.

## Materials and Methods

### Cell Culture, Transfections and Treatments

HEK-293 cells were maintained in Dulbecco's Modified Eagle Medium (DMEM) supplemented with 10% fetal bovine serum (FBS). hTERT RPE-1 cells were a kind gift of Dr. Gregory Pazour (University of Massachusetts Medical School, MA) and were maintained in DMEM/F12 media supplemented with 10% FBS. All transfections were performed using Lipofectamine2000 (Invitrogen) according to the manufacturers protocol. In order to obtain the HEK-293 cells in the quiescent phase, cells were serum starved for 16 hours in media lacking FBS, 6 hours after transfection. The quiescent cells were stimulated with 20% serum for an hour before harvesting. For hydrogen peroxide (H_2_O_2_) (Sigma-Aldrich) treatments, transfected cells were grown in normal growth media overnight (∼16 hours) and treated with 0.4 mM H_2_O_2_ for 1 hour before harvesting.

### Plasmid Preparation

The extracellular region of human CD44 with its single transmembrane domain (residues 1–289) was cloned in-frame with the C-terminus of polycystin-1 (residues 4106–4303) in pcDNA3 (Invitrogen) using the HindIII and ApaI sites. The control vector contained CD44 (residues 1–289) alone. N-terminal FLAG tagged CP1 was expressed in p3xFLAG-CMV (Sigma).

### Cell Lysates and Antibodies

Cell lysates from HEK-293 and hTERT RPE-1 cells were prepared in cold 1X cell lysis buffer (20 mM Tris (pH 7.5), 150 mM NaCl, 1 mM EDTA, 1 mM EGTA, 1% Triton X-100, 2.5 mM sodium pyrophosphate) containing 1X Complete protease inhibitor (Roche, Mannheim, Germany) and 1 mM Na_3_VO_4_. The lysates were analyzed by immunoblotting with the following antibodies: S6, phospho-S6 (S235/236), S6K, phosho-S6K (T389), AKT, phospho-AKT (S473), phosho-TSC2 (S939 and T1462), FoxO1 (L27), and phospho-FoxO1 (S256) (Cell Signaling Technology), LDH (Chemicon International), EGFP (Abcam), Flag (Sigma-Aldrich), GAPDH, lamin A/C (Santa Cruz Biotechnology, Inc.), TSC2 (Epitomics), beta-integrin (Clontech Laboratories, Inc.), TSC1 (Zymed Laboratories, Inc.). The E1/2 antibody is a mouse monoclonal antibody specific for the extracellular domain of CD44 and has been described previously [56]. Horseradish peroxidase (HRP)-conjugated goat anti-mouse, goat anti-rabbit and donkey anti-goat secondary antibodies were purchased from Santa Cruz Biotechnology, Inc.. All immunoblots were visualized using LumiGLO™ (KPL, Gaithersburg, MD) substrate.

### Human Tissue

All studies were conducted with specific approval of the Institutional Review Board according to NIH guidelines (categorized as exempt number 4) and in a HIPAA-compliant fashion. All specimens used in this study were designated as “discarded” and were supplied by a third party source (National Disease Research Interchange, Philadelphia, and the Polycystic Kidney Disease Foundation, Kansas) without identifiers.

### Immunohistochemistry

Age-matched (42–59yr) normal and ADPKD kidneys, perfused *in situ* and fixed at source in 4% fresh paraformaldehyde (EM Sciences, Hatfield, PA) in phosphate buffered saline (PBS), pH 7.4, embedded in paraffin were sectioned at 5 microns and subjected to immunohistochemistry using anti-phospho S6 (S235/236) antibody (Cell Signaling) and an indirect avidin-biotin-immunoperoxidase technique (Vectastain Elite, Vector Laboratories). Color development was carried out for 45 min using aminoethylcarbazole as substrate, generating a red reaction product. Tissue sections were mounted with Aquamount (Polysciences) and viewed under a Nikon Eclipse 2000 microscope equipped with Differential Interference Contrast (DIC) optics.

### siRNA Knockdown Assays and RT-PCR Analysis

Chemically synthesized siRNA SMARTpools were obtained from Dharmacon (human TSC2, M-003029-02; human PC-1, L-007666-00; siCONTROL, D-001210-01-05). The oligos were resuspended in 1X buffer to a concentration of 20 µM. The stock solutions of siRNA were diluted 1∶50 (making a final siRNA concentration of 20 nM) in 1X buffer, and DharmaFECT1 transfection reagent (Dharmacon) was diluted 1∶50 in OptiMEM medium (Invitrogen). The diluted siRNA and transfection reagent were then incubated for 20 minutes at room temperature, prior to transfecting the cells, in a 1∶2 ratio in a total volume of 300 µl. Knockdown efficiency was determined by RT-PCR analysis of mRNA collected from the cells 24 hours following transfection. mRNA was isolated from cells using the RNeasy™ Mini Kit (Qiagen), according to the manufacturer's protocol. mRNA was further purified by removal of DNA using Ambion's RiboPure™ Kit (Austin, TX), according to the maufacturer's instructions. Following RNA extraction, cDNA was made by reverse-transcribing 1 µg of RNA using the Invitrogen Superscript™ First-Strand Synthesis System for RT-PCR (Invitrogen, Carlsbad, CA). Aliquots of cDNA were made for each sample and stored at -20°C until analyzed. Real-time PCR was performed using the 7900T Fast Real-Time detection system from Applied Biosystems (ABI, Foster City, CA). Fast Real-Time Taq-Man assays from ABI were used to analyze gene expression of *PKD1*. All real-time PCR reactions were performed by mixing Universal Fast Real-Time Master Mix from ABI together with the gene assay mix first and then adding 2 µl of cDNA from each sample to make up a 25 µl volume. For an endogenous control, glyceraldehyde-3-phosphate (GAPDH) was used, which included probe and forward and reverse primer in a 25 µl reaction volume. The following set of conditions were used for each real-time reaction: 95°C for 10 minutes followed by 40 cycles of 1 second at 95°C and 20 seconds at 60°C. The real-time PCR reactions were all performed in triplicate and were quantified using the -ΔΔC_T_ method, which uses the average C_T_ of the GAPDH subtracted from the target gene C_T_ to obtain the average ΔC_T_. The control siRNA was used as a calibrator from which we subtracted individual PC-1 siRNA ΔC_T_ values to obtain the -ΔΔC_T_. The fold change for the sample was calculated in comparison to the calibrator by taking 2 ^−ΔΔCT^.

### Subcellular Fractionation

HEK-293 cells were harvested 24 h after transfection and washed with ice cold PBS. The cells were resuspended in hyotonic buffer (10 mM HEPES (pH 7.2), 10 mM KCl, 1.5 mM MgCl_2_, 0.1 mM EGTA, 20 mM NaF, 100 µM Na_3_VO_4_) and disrupted using a Dounce homogenizer. Crude nuclei were pelleted by centrifugation and the supernatant subjected to ultracentrifugation at 29,000 rpm at 4°C for 1.5 h. This yielded the cytosolic fraction (supernatant) and the membrane fraction (pellet). The pellet was lysed in 1X lysis buffer and the pure membrane fraction collected after centrifugation at 14,000 rpm for 10 min at 4°C. The crude nuclei were resuspended in hypotonic buffer and any unbroken cells disrupted in the Dounce homogenizer. The pellet after centrifugation at 3000 rpm at 4°C for 5 min was washed with a wash buffer (10 mM Tris (pH 7.4), 0.1% NP-40, 0.05% Na-deoxycholate, 10 mM NaCl, 3 mM MgCl_2_) and lysed in a high salt buffer (20 mM HEPES (pH 7.4), 0.5M NaCl, 0.5% NP-40, 1.5 mM MgCl_2_). The purified nuclear fraction was collected after centrifugation at 14,000 rpm at 4°C for 10 mins. The nuclear, cytosolic and membrane fractions were subsequently subjected to SDS-PAGE and immunoblot analysis.

### GST-14-3-3 Pulldown assays and Immunoprecipitations

GST-14-3-3 and GST (control) containing pGEX vector was overexpressed in *Escherichia coli* DH5α (Invitrogen) and the fusion proteins induced with 0.2 mM isopropyl–β-D-thiogalactopyranoside (IPTG). GST fusion proteins (GST alone and GST-14-3-3β) were then batch-purified from extracts, following sonication to lyse the cells, by binding to glutathione Sepharose 4B beads (Amersham Pharmacia Biotech) according to the manufacturer's instructions. The purified fusion proteins (10 µg) bound to agarose beads were mixed with 300-µg of protein from lysates of transfected cells and incubated overnight at 4°C. Beads were washed thrice in PBS containing protease inhibitors, eluted in 2x SDS sample buffer (125 mM Tris-HCl, 20% glycerol, 4% SDS, and 0.005% bromphenol blue), heated to 95°C, and analyzed by immunoblotting.

For immunoprecipitation analysis, the cell lysates from transfected HEK-293 cells were incubated with the indicated antibodies and protein A- and protein G – Sepharose beads (Amersham Biosciences, Piscataway, NJ) overnight at 4°C. The immunoprecipitates were washed with a wash buffer (10 mM Tris (pH 7.5), 1% NP-40, 1% Triton X-100, 100 mM NaCl, 50 mM NaF, 2 mM EDTA, 1 mM PMSF and Complete protease inhibitor (Roche, Mannheim, Germany)) and subjected to immunoblot analysis.

### Statistical Analyses

All statistical analyses were performed using the Student's T-test for determination of differences between the average values of quantitation data obtained from densitometric analyses of immunoblots. A value of p≤0.05 was considered to be statistically significant.

## Supporting Information

Figure S1 Immunocytochemistry (ICC) images at 40x magnification, showing staining for cilia using α - acetylated tubulin (cilia marker), and DAPI (nuclear marker) in HEK-293 and hTERT RPE-1 cells. The arrowhead indicates cells with cilia.
(0.59 MB PDF)
