# Supplementary figures and images for: Carboxy Terminal Tail of Polycystin-1 Regulates Localization of TSC2 to Repress mTOR
Source: PLoS One. 2010 Feb 16;5(2):e9239. doi: 10.1371/journal.pone.0009239 (PMC2821926; doi:10.1371/journal.pone.0009239)

$\alpha$ -acetylated tubulin

DAPI

Merge

HEK-293

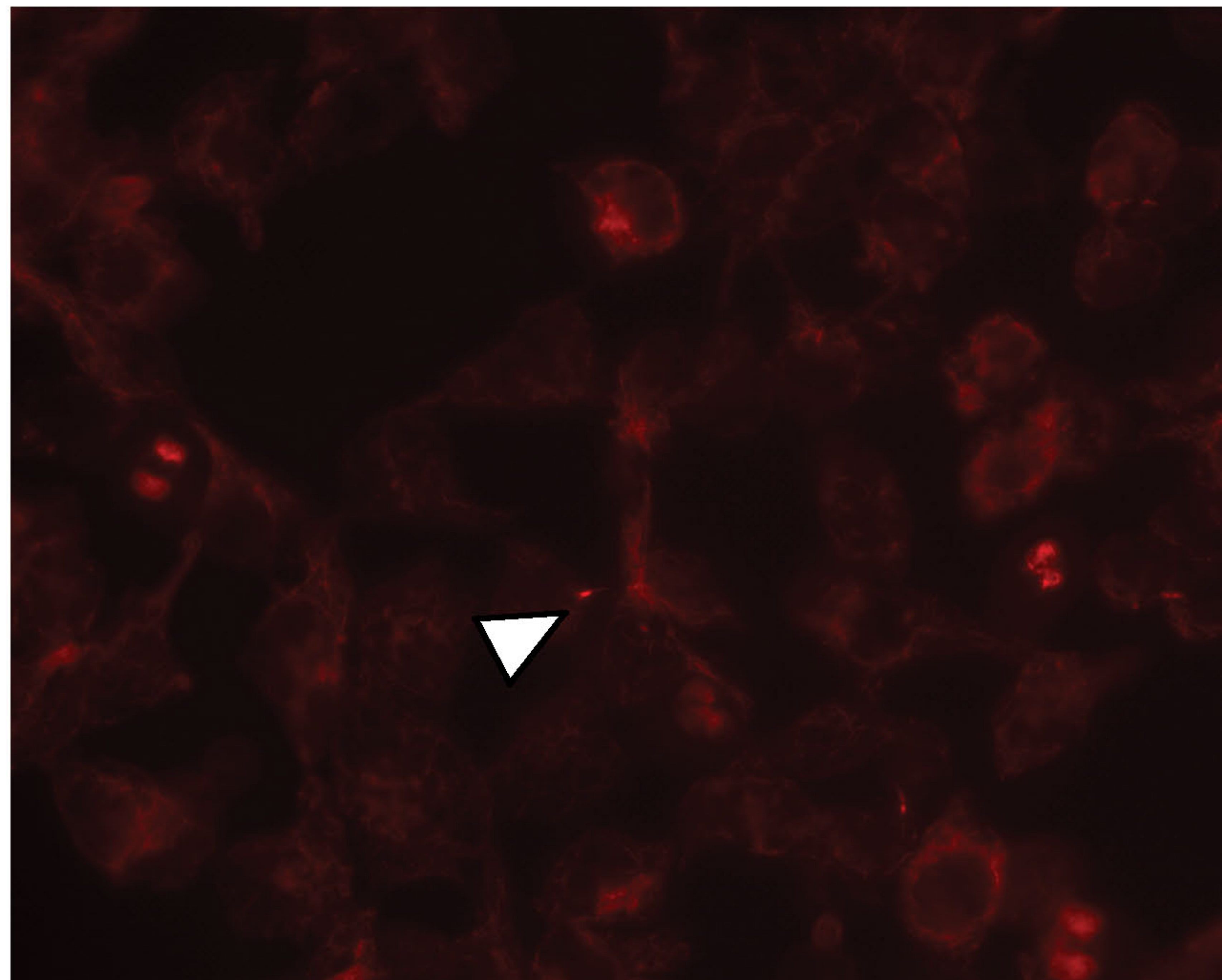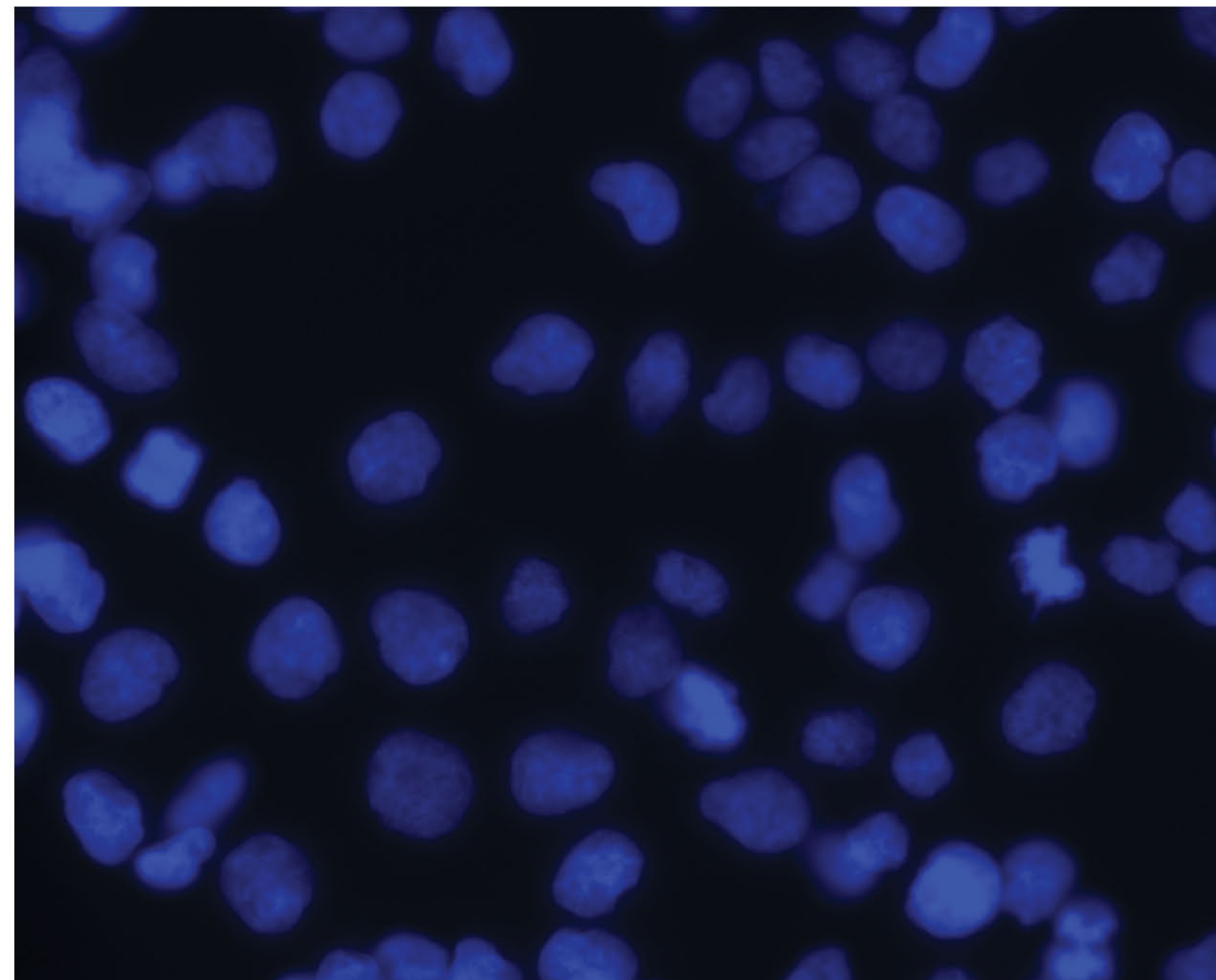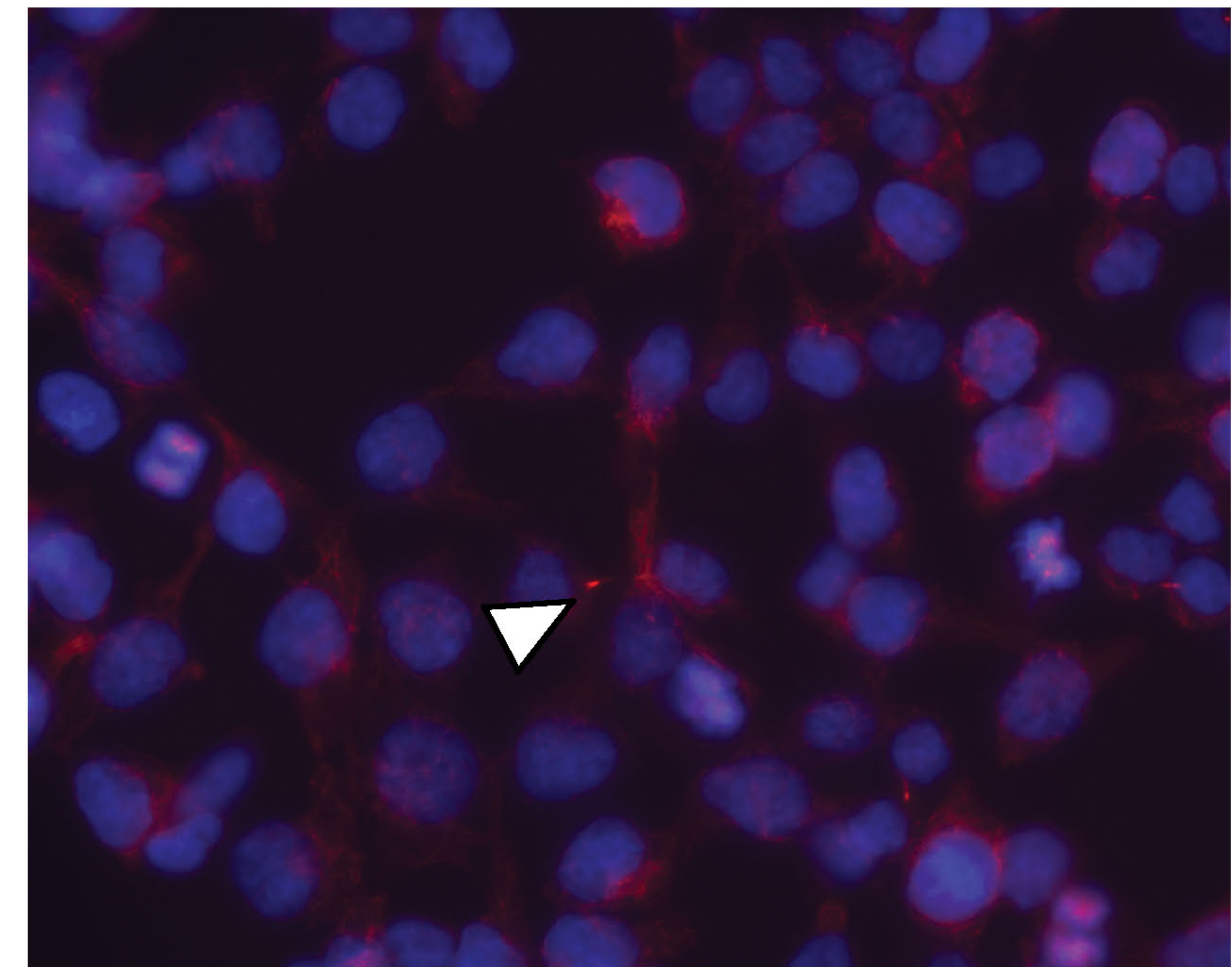

hTERT RPE-1

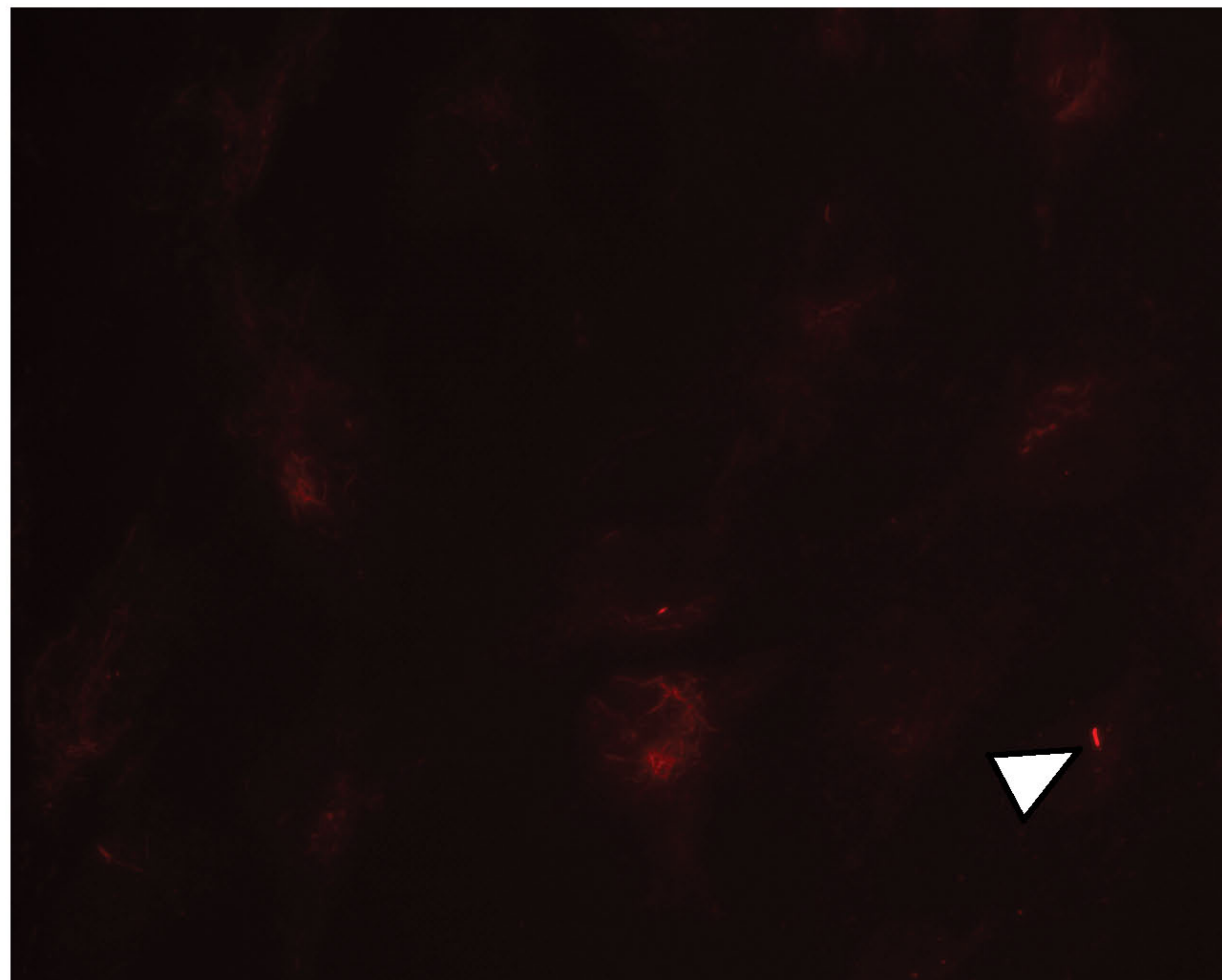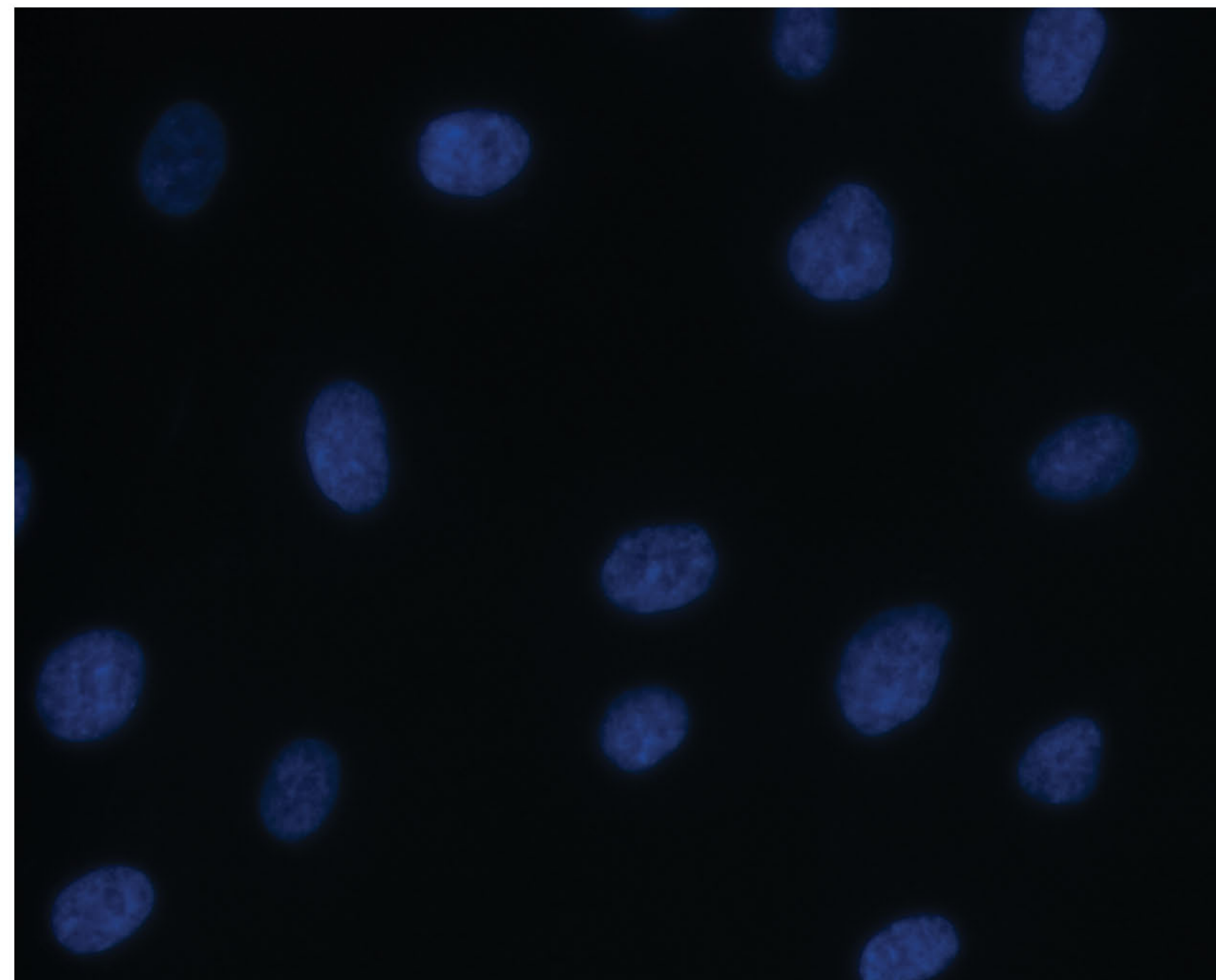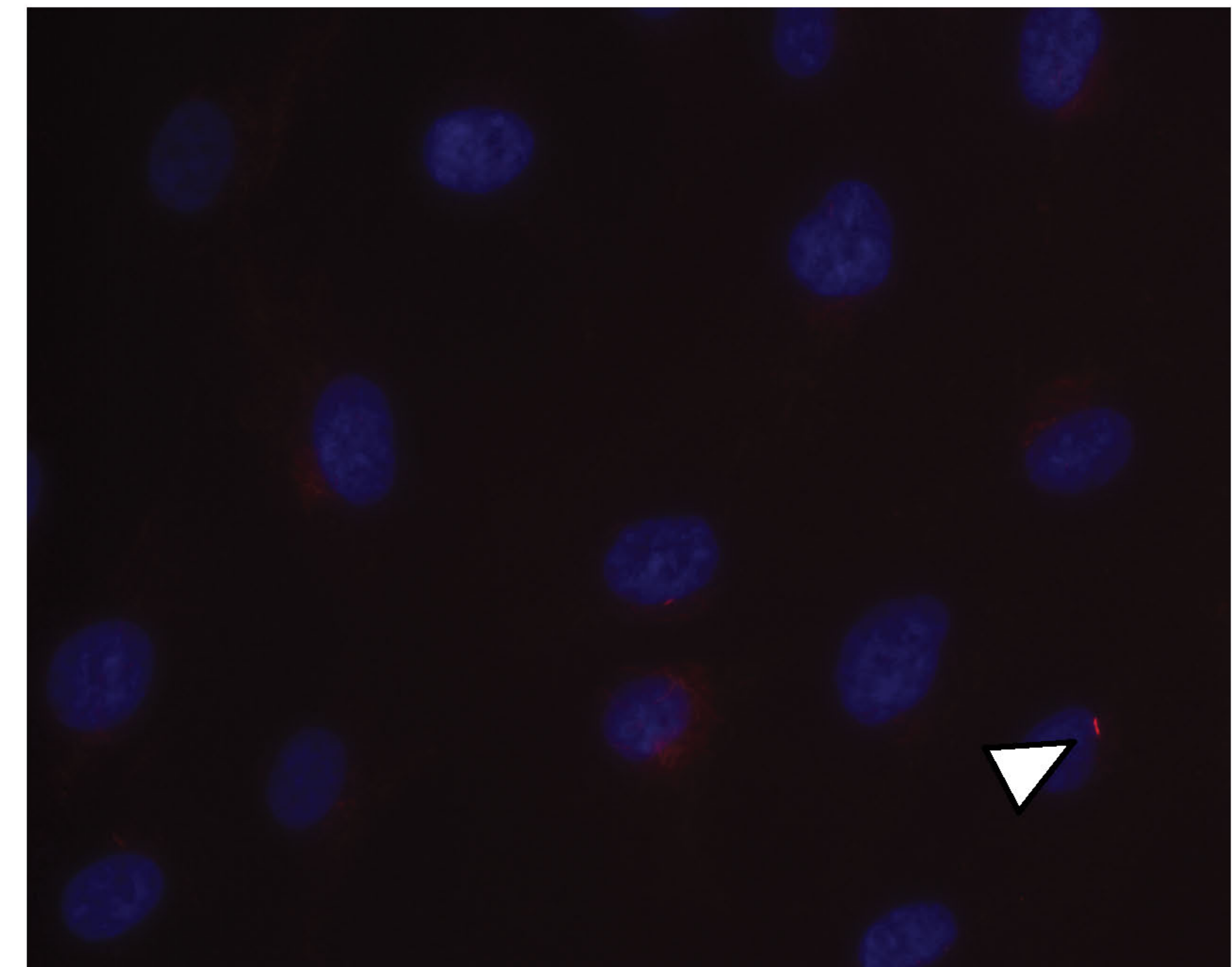

Supplement: Figure S1 — Immunocytochemistry (ICC) images at 40x magnification, showing staining for cilia using α - acetylated tubulin (cilia marker), and DAPI (nuclear marker) in HEK-293 and hTERT RPE-1 cells. The arrowhead indicates cells with cilia. (0.59 MB PDF) [file pone.0009239.s001.pdf]
